# The Well-being and Support Needs of Australian Caregivers of Neurodiverse Children

**DOI:** 10.1007/s10803-023-05910-1

**Published:** 2023-02-09

**Authors:** Emily D’Arcy, Tayah Burnett, Emily Capstick, Catherine Elder, Olivia Slee, Sonya Girdler, Melissa scott, Ben Milbourn

**Affiliations:** Curtin Scool of Allied Health, Curtin University, 6102 Perth, Western Australia

**Keywords:** Caregiver, Well-being, Neurodivergent, Mental Health, Support Needs

## Abstract

Caregivers of children with neurodiverse needs are known to experience challenges and hardship due to the increased needs of the child and the lack of support available. This study aimed to explore the support needs and well-being of caregivers of children with neurodiverse needs in Australia. Sixty-six caregivers participated in an online survey asking questions about support needs. The results highlighted five main themes that caregivers commonly experienced including: barriers to community engagement, impact on close relationships, negative impact on mental health and identity, financial hardship, and identified support needs. Findings identified multiple unmet needs existing amongst caregivers and further emphasises the importance of addressing these needs to improve the quality of life of caregivers of children with neurodiverse needs.

Difficulties associated with neurodivergent conditions generally emerge in early childhood and can remain prevalent throughout an individual’s life (American Psychiatric Association (APA), 2022; World Health Organization (WHO), 2018). Neurodivergent conditions manifest through cognitive, physical, behavioural, language, and social-emotional functioning impairments, which may impact social and occupational involvement (Mahone et al., 2017). These conditions can include but are not limited to; Autism Spectrum Disorder (ASD), Cerebral Palsy (CP), developmental delay, Attention Deficit Hyperactivity Disorder (ADHD), and learning disabilities (APA, 2022; WHO, 2018).

A child diagnosed with a neurodivergent condition often requires more support to engage in everyday activities than a typically developing child (Ritzema et al., 2017). If appropriate supports are not available, these support needs can bring about an array of challenges for caregivers that impact their health and well-being (Scherer et al., 2019). Meeting the child’s emotional, physical, social and learning needs, without adequate support, has been shown to result in an increased risk of caregivers experiencing elevated levels of stress, caregiver burnout and social isolation (Bozkurt et al., 2019).

Current literature has shown that caregivers of neurodivergent children experience a lower quality of life than caregivers of typically developing children (MacKenzie & Eack, 2022; Vaz et al., 2021). This lower quality of life is largely attributed to meeting the demands associated with providing ongoing care and support (Parsons et al., 2020). Current services for caregivers include caregiver training programs, education, support groups and respite (Lovell & Wetherell, 2019; Vaz et al., 2021). While there are many known services to support caregivers, knowledge of their specific effectiveness, relevance and accessibility are limited (Iadarola et al., 2018). The most frequently reported challenges as expressed by caregivers include a lack of social support, financial stress, fear for the future, and lack of parent education and training about accessing government funding and services (Daniels et al., 2017; Parsons et al., 2020; Vaz et al., 2021).

Meaningful relationships and social support for caregivers have been outlined in the literature as vital protective factors in reducing mental health concerns, decreasing psychological distress, and improving caregivers’ self-efficacy (Parsons et al., 2020). However, the impact of factors such as challenges in accessing services and the demands of navigating support systems on carer well-being and mental health has received limited attention (Pilapil et al., 2017). Caregivers of neurodivergent children are twice as likely to experience increased financial hardship compared to caregivers of typically developing children (Trentacosta et al., 2018). Research suggests that the more severe a child’s disability is, the more likely a family is to experience finance-related problems resulting from limitations in work capacity and the costs of therapy, adaptive equipment and assistive technology (Mohd Nordin et al., 2019; Nuri et al., 2019). Financial challenges increase the stress of these caregivers, with negative consequences for their quality of life and mental health (Vaz et al., 2021). Further, despite parents noting the benefits of training in managing their child’s behaviour and its positive impact on reducing parental stress and strain (Iadarola et al., 2018), the availability of this education is limited (MacKenzie & Eack, 2022).

The National Disability Insurance Scheme (NDIS) is Australia’s national disability support program providing “individualised support for people with disability, their families and carers” (National Disability Insurance Agency (NDIA), 2021). The scheme came into full operation in 2020, with the aim of providing reasonable and necessary supports for people with permanent and significant disabilities aged under 65 to improve their later outcomes in life (NDIA, 2021). The NDIS provides financial assistance for individuals based on their needs, including daily personal activities, mobility, transport, employment and education access, home and vehicle modifications and therapeutic support (NDIA, 2021). Given the recency of this scheme, it is not surprising that caregivers report significant levels of difficulty in accessing and navigating this system (Gavidia-Payne, 2020; Olney & Dickinson, 2019).

Neurodivergent children and their families represent the largest single group of participants accessing the NDIS (NDIA, 2021). While the NDIS has many benefits, it places significant responsibility on caregivers, particularly in advocating for their children and managing their plans. Families have also expressed some frustration that the scheme is tailored towards individual adults instead of families and carers (Russo et al., 2021). Collectively this body of evidence suggests that there are significant gaps in meeting caregivers’ support needs and well-being needs. Therefore, this study aims to explore the support needs and well-being of Australian caregivers of neurodivergent children to inform service development and policies concerning the best approaches to supporting caregivers of neurodivergent children.

## Methods

### Design

This study was guided by an exploratory multiple method design utilising a survey approach (Creswell et al., 2003). Standardised measures of caregiver stress and well-being were employed combined with multiple-choice questions and open-ended qualitative questions (Liamputtong, 2020). This was considered a suitable approach as it enabled researchers to gain an in-depth insight into the experience of caring for neurodivergent children (Mayoh & Onwuegbuzi, 2015).

### Recruitment and Sample

A purposive non-probability sampling method was used to recruit participants, which allowed access to individuals that met the inclusion criteria for the study (Palinkas et al., 2015). Recruitment took place between May and June 2021, focusing on recruiting caregivers of a child/children with a diagnosis of a neurodevelopmental disability between the ages of 0–12, and currently living in Australia. Participants were recruited via advertising to multiple disability non-government organisations, carer social media platforms, and word-of-mouth. No incentive was provided for caregivers who completed the survey. A total of 66 participants completed the survey, with demographic characteristics displayed in Table 1. Most participants were female (n = 63, 95.5%), with a mean age of 39.7 years. The majority of the sample was from Western Australia (n = 52, 78.7%). Fifty participants were married (75.8%). Twenty-eight participants (42.4%) worked part-time, and nineteen participants (28.8%) were not studying or working part-time, full-time or casually outside the home. Participants cared for two children (both neurotypical and neurodivergent) on average. The children’s ages ranged between 2 and 22 years, with 68.1% (n = 62) being male. The average age of the neurodivergent children was 8.1 years. The diagnoses of participants’ child/ren included ASD (n = 63, 68.1%) and ADHD (n = 24, 25.3%). Neurodivergent children had an average of 1.78 diagnoses (Range: 1–7).

**Table 1:** Demographic information of participants (N = 66)

Characteristic		n	%	Mean	SD	Range
Gender	Male	3	4.5			
	Female	63	95.5			
Age (years)				39.7	6.6	25–53
Relationship Status	Married	50	75.8			
	Divorced	3	4.5			
	De Facto	6	9.1			
	Separated	3	4.5			
	Never Married	2	3.0			
	Other	2	3.0			
Level of Education	Less than a high school diploma	2	3.0			
	High school Year 12 or equivalent	8	12.1			
	Certificate III, IV, or advanced diploma	22	33.3			
	Bachelor’s degree (e.g. BA, BS)	20	30.0			
	Graduate Certificate	1	1.5			
	Graduate Diploma	3	4.5			
	Master’s Degree	8	12.1			
	Doctoral Qualification (e.g. MD, PhD)	1	1.5			
	Other	1	1.5			
Work	Full time	7	10.6			
	Part-time	28	42.4			
	Casual	7	10.6			
	Study full-time	0	0			
	Study part-time	9	13.6			
	Nil work/study	19	28.8			
Neurodiverse	Yes	14	21.2			
	No	49	74.2			
	Prefer not to say	3	4.5			
Number of children				2.3	1.1	1–8
Relationship with children	Biological	64	97.0			
	Foster carer	1	1.5			
	Other	1	1.5			
Age of children (years)				8.1	3.8	2–22
Gender of children	Male	62	68.1			
	Female	24	26.3			
	Non-binary	2	2.2			
	Missing	3	3.3			
Diagnosis of child	Autism Spectrum Disorder	63	69.2			
	Attention Deficit Hyperactivity Disorder	23	25.3			
	Global Developmental Delay/Developmental Delay	7	7.6			
	Anxiety	6	6.6			
	Speech Language/Disorder	6	6.6			
	Oppositional Defiance Disorder	5	5.5			
	Sensory processing Disorder/Profile	5	5.5			
	Intellectual Disability	4	4.4			
	Specific Learning Disorders	2	2.2			
	Movement Disorders (e.g. CP, DCD)	3	3.3			
	Other (e.g. genetic conditions)	11	12.1			
	None (sibling)	3	3.3			
	Not given or unclear	10	10.1			
Number of diagnoses				1.78	1.2	1–7

### Survey

A secondary analysis of previously collected qualitative interview data, with four caregivers exploring the experiences of caregivers of neurodivergent children, informed the online survey questions. This analysis provided background to this research, including identifying common contributors to carer well-being, including the impact of social support, societal stigma, diagnostic process, mental health and well-being, and education on caregivers. The “Caregiver Well-Being Survey” consisted of a maximum of 114 questions, where the number of questions presented depended on branching from previous answers and the number of support need areas the caregiver wished to describe (the survey is provided in Supplementary File A). Context was provided using quantitative multiple-choice questions before qualitative questions were asked to gain further insight into the experience. The survey was created on Qualtrics software. Questions comprised of dichotomised questions, four-point Likert-type scales, multiple response options and short answer questions. Questions covered the following topics: (1) Demographic information (age, gender, relationship status, highest level of education and current employment status); (2) Caregiver experiences and impact on well-being, and (3) Caregiver specific support needs. The Caregiver experiences and impact on well-being section included the 12 questions from the Personal Well-being Index; (PWI), (International Wellbeing Group, 2013., pp 16–17), focusing on life satisfaction. Within the support needs section of the survey, caregivers identified and described the specific supports they accessed, including the number of support need areas. After the sections of the Caregiver Well-Being Survey were administered, participants were asked if they were willing to complete the Parent Stress Index- Short Form (PSI-SF; Abidin 2012), if so, the questions from the PSI-SF were administered within Qualtrics. The survey was piloted with two parents prior to distribution, and minor changes were made to the survey based on their recommendations.

### Data Collection

Participants interested in completing the survey were provided with an online Qualtrics link (Qualtrics, 2021). Participants completed the survey anonymously to facilitate participants expressing their authentic experiences and thoughts. The estimated time of completion for the survey was 50 min. The online format of the survey allowed caregivers with multiple demands and those living anywhere in Australia to participate. This increased the participant pool and strengthened the study’s validity (Uhlig et al., 2014).

### Data Analysis

The online responses to the survey were exported from Qualtrics and converted to a Microsoft Excel spreadsheet for cleaning, coding, and analysis. Descriptive statistics (percentiles, counts, averages, standard deviations, medians and ranges) were used to summarise participants’ demographics, caregiver experiences and the need for caregiver-specific supports (Portney & Watkins, 2015). The PSI-SF and PWI were scored according to their respective manuals and compared to the available Australian normative values (Abidin, 2012; International Wellbeing Group, 2013). Caregivers were also asked to score the impact of being a caregiver for a neurodivergent child for each component of the PWI.

Qualitative analysis of the responses to open-ended questions was developed using Braun and Clarke (2006) thematic analysis six-step process. This process consists of (a) familiarising oneself with the data, (b) generating initial codes, (c) searching for themes, (d) reviewing themes, (e) defining and naming themes and (f) producing the report (Braun & Clarke, 2006). Data was transcribed into a word-processing document and exported to Nvivo, which was used as the tool for analysis during the generation of initial codes and searching for themes (Q R S International Pty Ltd, 2020). Initial coding was independently undertaken by four authors (TB, EC, CE and OS), resulting in the generation and labelling of unique codes for each relevant response. The remainder of the authors (BM, ED SG, MS) then reviewed the coding and labels. Agreement on codes was discussed over several meetings and after a review of the full dataset, codes were then subsequently organised into themes by the members of the team (BM, ED, SG, TB, EC, CE, OS). Each theme was reviewed and discussed for consensus agreement amongst the research team. The trustworthiness of open-ended responses was assessed using the following criteria: credibility, transferability, dependability and confirmability (Guba & Lincoln, 1989). To address credibility, members of the research team spent a period of weeks reading, reflecting and rereading the data. To address transferability a thick description of the data has been provided. Dependability involved the development of a clear audit trail in terms of describing the methods used and clearly presenting the findings using quotations to demonstrate themes. Senior research team members also double-checked the coding trail to confirm the themes identified in the initial coding cycle. No discrepancies were observed between the three reviewers in the identified themes. All researchers convened to discuss the codes and categories determined during the first coding cycle. Confirmability was addressed by ensuring a clear presentation of participant responses and by providing a clear rationale for each step involved in the methods and analysis.

### Ethical Considerations

Ethical approval for this study was granted by Curtin University Human Ethics Committee in May 2021 (Approval number HRE2019-0001-25). Consent was gained through the survey from each participant prior to data collection. The research complied with the requirements of the National Australian Data Storage requirements (The National Health and Medical Research Council, 2019).

## Results

### Caregiver Responsibilities

As shown in Table 2, participants, on average, cared for their neurotypical child/ren for 14.5 h (SD = 7.8) and their neurodivergent child/ren for 16.3 h (SD = 6.6). The participants’ primary carer roles included daily routine (n = 66, 100%), mealtimes (n = 65, 98.5%), organising health appointments (n = 65, 98.5%) and safety and protective behaviours (n = 65, 98.5%). Less common carer roles included school access (n = 47, 71.2%), and community access (n = 52, 78.8%).

**Table 2:** Caregivers’ Experiences

		n	%	Mean	SD	Range
Average hours caring for neurotypical child/children				14.5	7.8	2–24
Average hours caring for neurodiverse child/children				16.3	6.6	3.5–24
Receiving financial support*	Yes	24	52			
	No	22	48			
Caregiver NDIS funding*	Capacity Building Supports	3	100			
	Core Supports	3	100			
	Capital Supports	2	66.7			
Gaps in Caregiver NDIS funding*	Yes	3	100			
	No	0	0			
Child/ren’s NDIS funding*	Capacity Building Supports	32	89			
	Core Supports	32	89			
	Capital Supports	7	19			
Gaps in Child/ren’s NDIS funding *	Yes	26	70.3			
	No	11	29.7			
*Percentages calculated from the number of responses						

### Caregiver Supports

**Fig. 1:**
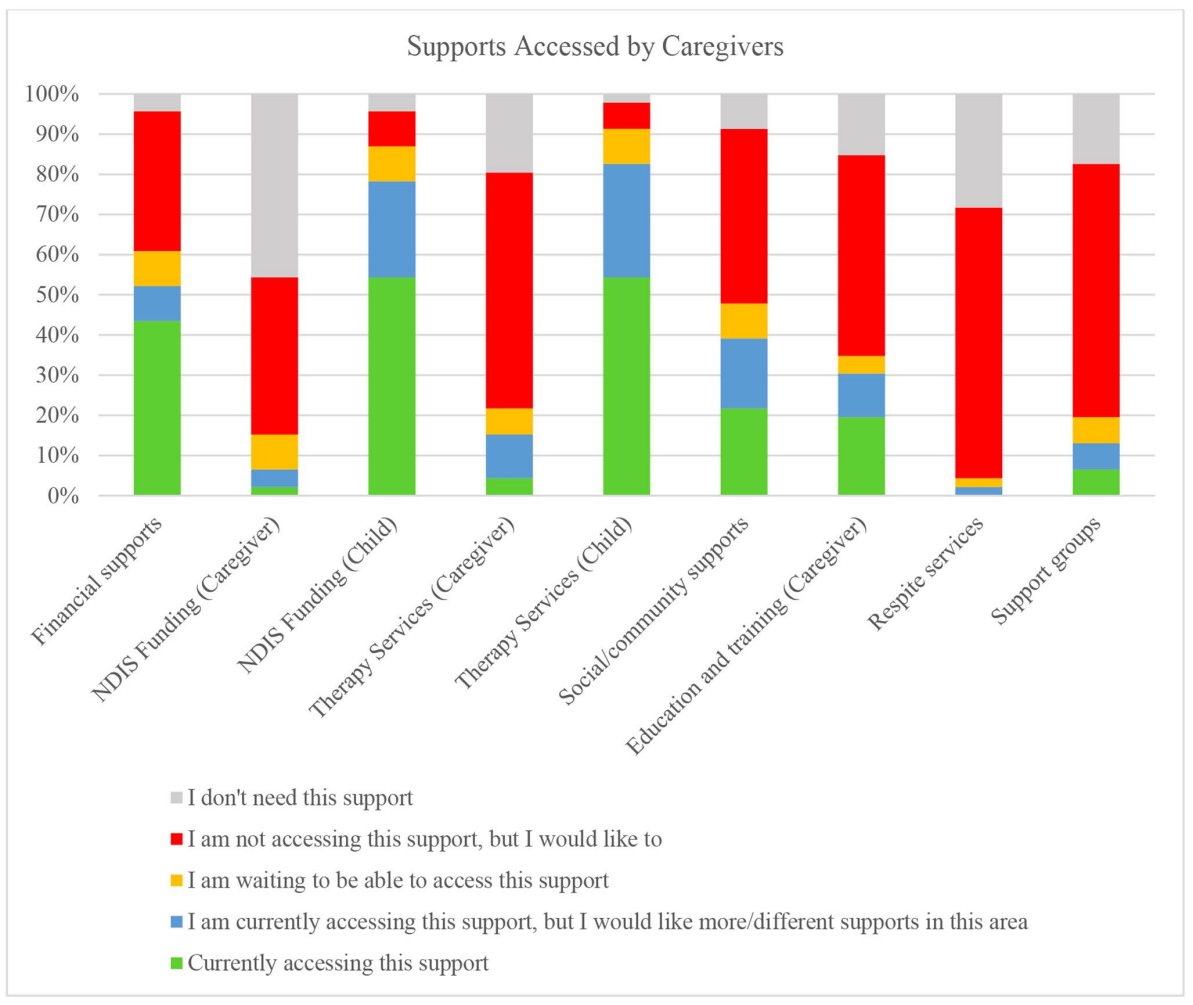
Supports Accessed by Caregivers

The participants’ responses revealed that 34% (n = 16) were currently not accessing carer payments but would like to (Fig. 1). The survey reported that 53.2% (n = 25) of participants were currently accessing funding for their neurodivergent child/ren through the NDIS, of which 70.3% (n = 26) reported gaps in their child’s funding. Most caregivers’ children received capacity building (those to develop skills, such as occupational therapy) and core supports (those to facilitate participation, such as transport costs; n = 32, 89% for both). Only 19% (n = 7) of caregivers were accessing capital supports (high-cost assistive technology, equipment and home or vehicle modifications) for their child(ren). Concerning support needs (as shown in Fig. 1), approximately half of the participants reported they were not accessing services but would like to be, including therapy services for themselves (n = 28, 59.6%), social/community support (n = 21, 44.7%) and education and training for themselves (n = 24, 51.1%). None of the participants were currently accessing respite services, and only three participants (6.4%) were currently accessing support groups.

### PWI and PSI-SF Scores

Across the sample, the mean total score of the PWI was 54.8 (SD = 21.8) compared to the National Subjective Well-being Measures (2018) mean of 75.1. Of the 66 participants, 82% (n = 54) had a total PWI score lower than the national mean. The areas of lowest satisfaction across the sample were *your health* (*M* = 41.7, *SD* = 24.7) and *feeling part of your community* (*M* = 48.6, *SD* = 27.2). While the areas of highest satisfaction were *how safe you feel* (*M* = 64.1, *SD* = 25.9) and *standard of living* (*M* = 61.4, *SD* = 23.1). On average, the participants reported a moderate satisfaction rating in most areas of the PWI. However, participants reported exceptions including *how safe you feel* as the least impacted area (*Mdn =* 1.5). *Life as a whole* was the area with the greatest percentage of complete impact scores (n = 27, 41%).

On average, the caregivers scored in the 91.7th percentile (SD = 9.9) on the PSI-SF’s total stress score, indicating clinically significant stress levels (under standardised testing conditions, scores above the 90th percentile are clinically significant, and scores over the 81st percentile are ‘high stress’). More specifically, within the PSI-SF, on average the sample fell in the 97th (Total score 44.5, SD 8.4) percentile for the *difficult child* domain, referring to how a parent perceives how difficult his/her child to be. Within the domain of parent-child dysfunction interaction, caregivers scored within the 96th percentile (*M* = 39.8, *SD* = 8.8), and within parenting distress, they fell within the 92nd percentile (*M* = 40.6, *SD* = 8.8).

### Qualitative Themes

The following themes were described by participants as being both an impact on their well-being and a support need, and although they are identified as distinct themes alone, many of these ideas are interconnected.

### Barriers to Community Engagement

Caregivers described experiences of judgement from people in Australian society due to preconceived negative stereotypes and stigmas associated with their child’s challenging behaviours. These negative stereotypes impacted the way caregivers interacted in their community. For example, one participant described how they felt: “*very restricted in how we can be part of our community.“.* One participant’s depiction of the judgement held by society and the consequential emotional toll which is placed upon caregivers: “*it makes it extremely difficult and emotionally confronting to get out with my kids. People are so judgemental about the smallest things.“*. Participants further expressed the isolation experienced as a result of their reduced community engagement, as one participant described: “*Sometimes we avoid things – going out in the community or attending things because of the needs of our neurodiver[gent] child”.* Participants also noted a significant societal lack of understanding of neurodivergent conditions, resulting in social isolation and decreased emotional well-being.

### Impact on Close Relationships

Participants expressed that their role of being a caregiver for their neurodivergent child had a negative impact on the relationships in their lives, including family, friends, and partners. Participants described how they found it immensely difficult to socialise with friends due to the lack of time available, as their child’s needs would take priority. Many reported feeling socially isolated with no one to reach out to for support. Participants also related this feeling of isolation to a lack of understanding shown by parents of neurotypical children. Participants reflected how they often felt excluded and isolated from social circles due to their attitudes towards their child’s behaviours or diagnosis. One participant disclosed: *“I experienced a loss of friends, as if what my child has is an infectious disease”*. Participants expressed these feelings led them to feel alone and struggling to cope with the overwhelming challenges that come with caring for a neurodivergent child.

Participants also described the impact that being a caregiver has on the relationship with their partner. Participants frequently reported having limited time to spend with their partner due to their shared parenting role. The overwhelming schedule and workload they are faced with to meet their child’s needs made spending quality time together difficult. Participants related this to not having enough external support at home to be able to fulfil their role as parents and meet the needs of their child, as well as being able to spend time as a couple: *“My husband and I treat parenting like shift work in order to get respite so there are limited opportunities for quality parent time”*.

### Negative Impact on Mental Health and Identity

Participants’ narratives reflected that the role of a carer contributed to a negative impact on their mental health. A participant described, “*my mental health has suffered significantly. I am under constant strain focusing everything on his needs and well-being”.* Burnout and exhaustion were reported by participants in relation to the frequent appointments, extensive waitlists and difficulties communicating with the NDIS and support agencies, difficulties managing their neurodivergent child’s behaviour and the challenges associated with maintaining a regular schedule. Participants described the “*huge stress and frustration”* associated with managing waitlists, appointments, and the NDIS system expressing:

“*The NDIS is a poor system with too many middle man positions taking money and is not serving people with disabilities well or cost effectively. It has given my child and myself more headache than help since being accepted onto the scheme.“*

Participants have described the inability to develop a routine as being “*mentally and emotionally exhausting”.* They also outlined the exhaustion associated with caring for their child, which contributes to their depression and isolation.

Participants articulated a sense of loss in self, identity and career opportunities. This loss was reported to be primarily due to restricted work hours and limited employment opportunities due to increased hours caring for their neurodivergent child: “*my career is in jeopardy as I am unable to work fulltime”.* Participants discussed experiencing burnout, stress, and being overwhelmed, contributing negatively to their personal sense of well-being and employment capacity: “*I have become so accustomed to focusing on my child’s needs that I have forgotten how to follow through on my aspirations.“.*

### Financial Hardship

Participants openly discussed experiencing financial hardship primarily due to their limited earning capacity and additional expenses associated with caring for a neurodivergent child. For example, as described by one participant:

“*I am faced with the choice of staying home, having the time to assist them [neurodivergent children] but not enough money to continue their treatments, or returning to work to fund the therapies they need to learn to live in a neurotypical world, but not having the time to support them.“.*

Many of the additional supports caregivers wish to access for their children are not covered under the NDIS, such as respite and social and community access. Therefore, they are required to either forgo the support or fund it themselves. The high cost for additional supports were reported to contribute to the financial hardship experienced by participants. Participants consequently expressed difficulty accessing and managing their NDIS plan to ensure they had the appropriate funding to support their child’s needs. The significant cost throughout the diagnostic process was also reported to contribute to the financial hardship participants experienced. Single-income caregivers, particularly, noted financial hardship and were forced to make career sacrifices in order to meet the needs of their neurodivergent child.

### Identified Support Needs

Participants from the study identified an array of areas in which they require support including support groups, education and training, respite, and financial assistance. Caregivers expressed the need to be connected to other caregivers to provide them with the opportunity to share lived experiences and parenting advice. Participants highlighted the need for these support groups to be easy to find, join, and attend. Participants also identified parent training and education as a support need, particularly the need for caregivers to be educated on how to manage their child’s challenging behaviours and best support their neurotypical children. Education about their child’s condition was identified to be required for society to be provided knowledge to reduce judgement experienced by caregivers. Community education was described as enhancing care provided to their neurodivergent children in schools and facilitating positive community experiences. This was identified in conjunction with the need for respite. For example, one participant described: *“I’m not able to do activities I would like because I am unable to find appropriate care/take him with me because he will be distressed.”* Participants identified the need for respite to have more time to focus on themselves, their mental health and their well-being. Specifically, to be able to assist them in dedicating time for their relationships with family and friends, and extra time to organise and structure their lives. Participants reported the need for additional funding to access support services, including psychology/counselling, resources/education, and home maintenance. Participants described the need for shorter waitlists, decreased cost of diagnosis process and subsequent therapeutic interventions to assist them financially. The support needs identified were expressed by participants to enhance the quality of care provided to their child as well as positively improving their personal well-being through reduced stress.

## Discussion

This study sought to explore the well-being and support needs as expressed by caregivers of neurodivergent children. Results indicated poor well-being, high levels of parent stress and poor mental health. This is consistent with studies in similar populations, such as Friesen et al. (2021) which identified significant levels of mental distress and carer burnout in caregivers of neurodivergent children in Canada. These results in the current study identify a clear need for caregiver mental health support within the Australian community. This is of particular import, as the consequences of continual caregiver stress can have long-lasting psychological and emotional effects (Manning et al., 2021). Alongside child-specific variables, lack of supports and the stress of advocating for support for their child are some of the main predictors of caregiver burnout (Shepherd et al., 2018). Caregivers from this study articulated that their greatest stressors were around the need for respite and a lack of support from family, friends and services. This need for supports to reduce stress and exhaustion can often reach desperate levels (Teo et al., 2018). Lack of appropriate and sufficient supports not only adversely impacts caregiver well-being but can also contribute to poorer outcomes for their child, including behavioural and emotional difficulties (Trentacosta et al., 2018).

Perceived social support has been shown to serve as a protective factor influencing the relationship between child behavioural and emotional difficulties and parental life satisfaction (Halstead et al., 2018). The benefits of support programs include enhanced well-being of caregivers, attributed to the improved understanding of their child’s condition, feelings of hope throughout the group and realisation they were not alone in their experiences (Zuurmond et al., 2019). Caregivers’ skills including advocating for their child have the potential to have a positive influence in supporting vulnerable caregivers (Lee et al., 2022). However, participants of this study identified a lack of accessible support groups, and a lack of education and training for supporting their child. They also noted that support groups were difficult to find, join and attend. This suggests there is a need for caregiver support groups to be more accessible for caregivers and their unique needs (Pilapil et al., 2017).

Results from this study indicated a significant number of caregivers reporting the negative stigma and stereotypes which exist in their community about their neurodivergent child. The negative stigma was expressed to hinder their engagement with the community, with 22.7% of participants not feeling part of a community, thereby exacerbating their feelings of isolation. Caregivers of this study felt stigmatised during community outings and interactions, which is consistent with Liao et al. (2019) who found that these feelings from parents were related to the perceived stigma associated with their child’s behaviour. This stigma occurs in addition to the consistent demands and pressures affiliated with raising a neurodivergent child, which results in them withdrawing from their community to avoid stigmatisation (Broomhead, 2019; Papadopoulos et al., 2019). Future caregiver interventions and supports should specifically focus on protecting caregivers from the existing stigma, until the prevalence of public stigma is significantly reduced (Papadopoulos et al., 2019). This can occur in the form of providing caregivers with practical skills to utilise in future community interactions including; self-compassion, mindfulness techniques and, pre-developed responses to negative stigma encounters (Papadopoulos et al., 2019). Also important is educating the general public about what stigma is, how caregivers experience it, and how the general public is contributing to negative and stigmatising experiences (Mazumder & Thompson-Hodgetts, 2019). Implementing stigma-directed interventions is essential to increase community knowledge and awareness (Chan & Lam, 2018; Liao et al., 2019). This may occur by improving community partnerships and implementing techniques to upskill the community (Kuzminski et al., 2019).

Caregivers of this study explored the financial stress associated with the challenges of maintaining employment or being on a single income. Results indicated that just under half of participants are currently accessing a range of carers payments, a common occurrence in this population in order to support their family and access services for their child (Marsack-Topolewski, 2020). Raising a neurodivergent child is more financially costly in comparison to raising a neurotypical child, with average costs to Australian families between $33,000 and $97,000 per annum above typical child-related costs (Arora et al., 2020; Baker et al., 2021; Horlin et al., 2014). The majority of this cost is associated with reduced employment in order to support the needs of their neurodivergent child (Baker et al., 2021; Horlin et al., 2014). The financial strain this study’s caregivers experienced was also attributed to the high costs of the diagnosis process and accessing intervention and other support services. Hence, it appears more financial support is required to assist in these areas of supporting a neurodivergent child (Reddy et al., 2019).

Caregivers of this study were frequently required to make personal sacrifices concerning their career and employment options, which negatively influenced them financially and their personal identity. While 95.6–97.9% of at least one partner of couple families with dependents in Australia are employed, just under half of carers are working in a paid job, and of those unable to work, 85% reported their child with a disability as an identified barrier to working (Australian Bureau of Statistics, 2022; NDIA, 2021). Comparatively, participants of this study saw 63.6% of carers employed (full-time, part-time or casually) and indicated the implications on their work capacity due to the lack of flexibility involved with being a caregiver and employers. Caregivers are faced with the obstacles of succeeding in their work environment or finding appropriate childcare services that cater to their neurodivergent child’s needs (Eskow & Summers, 2019). The inability to succeed in their career negatively influences their personal identity (Schertz et al., 2020). This impaired sense of identity can be attributed to caregivers’ perceived lack of control, reducing their sense of well-being (Cooke et al., 2020). Therefore, the need for caregivers’ sense of identity and well-being to be supported by services across the health and disability sector is evident.

The frustration and emotional challenges associated with the diagnostic process were another key theme in these results. These challenges included the long waitlists to receive their child’s diagnosis, lack of information once they have received their child’s diagnosis, and difficulties managing and accessing funding through the NDIS. Currently, in Australia, caregivers can expect a waitlist of at least ten months to commence the process for an autism diagnosis (Smith-Young et al., 2020), despite the identified need for delivering timely and accurate diagnoses (D’Arcy et al., 2021). This includes evidence-based, professional education and training to caregivers, including relating the early signs of their child’s diagnosis (Bent et al., 2020). Currently, while awaiting assessment and diagnosis for their neurodivergent child, caregivers are not provided with support or information to assist them (Creen et al., 2021). This lack of support was found to negatively impact caregivers’ mental health and well-being, as they experience difficulties knowing how to best support or understand their child’s behaviours. Coaching via telehealth during the waitlist time has been demonstrated to effectively enhance parent’s utilisation of intervention strategies for challenging behaviours for toddlers awaiting diagnosis of autism, which in addition, improves caregivers’ self-efficacy and quality of life (Carey et al., 2021; Kunze et al., 2021). Once receiving a diagnosis for their child, caregivers from this study responded with confusion and high levels of stress, similar to those identified by Evans et al. (2022). To address the after-effects of a diagnosis, it may be beneficial for caregivers to be offered counselling or linked to support groups to assist with their emotional processing of their child’s diagnosis (Evans et al., 2022). In addition, caregivers from this study required more detailed information from health professionals regarding their child’s condition, reason for behaviours, and strategies to best support their child.

There are under half a million participants across Australia part of the NDIS, and an additional 13,400 as part of the Early Childhood Early Intervention (ECEI) Gateway (ECEI; NDIS Quarterly Reports to disability minister, 2021). The NDIS has reportedly improved 75% of carers’ access to services, programs and activities in the community and improved the health and well-being of approximately half of carers of children aged 0–14 years. However, participants of this study still indicated gaps in their child’s NDIS funding. This may be related to the challenges families experience navigating the complex NDIS system, which is a process still misunderstood by many (Lakhani et al., 2018; Gavidia-Payne, 2020). Originally created for adults with a disability, the system’s design creates an ineffective support system for Early Intervention clients (Carey et al., 2021). Consequently, caregivers whose children were involved in the NDIS ECEI gateway experienced increased mental health concerns, similarly to the participants of this study (Gavidia-Payne, 2020). There is also a risk that children will not receive the full level of support offered by the system, further impacting caregivers’ well-being and stress. Caregivers need to be supported and educated by individuals with excellent knowledge of the NDIS who can provide caregivers with support and advocacy (Perry et al., 2019). This would likely assist caregivers in understanding how to best navigate the NDIS and maximise their child’s therapy plan, to ensure the best outcomes for both the child and caregiver (Carey et al., 2021). While there are some resources available to support caregivers in this way (e.g. Family Advocacy, n.d), there appear to be barriers preventing caregivers from benefiting from these services which should be further explored.

### Limitations

Given this study’s sample size, the responses provided by participants do not adequately represent the experiences of all caregivers of neurodivergent children. The study’s design may also limit the generalisability of the findings to the broader neurodivergent population. Future studies should attempt to capture a more heterogeneous sample, including caregivers from culturally and linguistically diverse backgrounds, and caregivers from regional and remote Australia. However, the target population of this study is particularly hard to reach, given their vulnerability and the sensitive nature of the study (Liamputtong, 2007). Due to the demands of participating in research, it could be assumed that the participants who agreed to complete the survey are caregivers with less severe difficulties, therefore, results may not reflect the most severe experiences of caregivers. Due to the majority of the sample being biological mothers, the results are limited to the experiences of caregiving from a mother’s perspective. While the use of a survey in this study was advantageous in reaching a large population of caregivers due to COVID-19 restrictions and the limited time that caregivers have, further research consisting of in-depth interviews would be beneficial in gaining a more extensive understanding of caregiver experiences. Additionally, only a small number (5%) of this study’s caregivers received supports for themselves through the NDIS, and so further research is required with a larger sample to explore the experiences and support needs of caregivers of neurodivergent children who have NDIS plans of their own. Nevertheless, the findings provide insight into the experiences and well-being of caregivers of neurodivergent children, and indicate the need to co-produce research with caregivers, to better inform lived experience (Rycroft-Malone et al., 2016).

## Conclusion

This study explored the different types of support needs and the impacts of caring for a neurodivergent child on caregiver well-being. Findings indicate that there is an array of unmet needs amongst this population, including support groups, education and training, respite and financial assistance. Understanding their collective experiences indicates that there are significant negative impacts on caregiver health, including clinically significant higher levels of stress, social isolation, and caregiver burnout, all contributing to significantly reduced personal well-being. Significant time and effort must be placed into developing more accessible services and increasing caregiver understanding and education, to improve the well-being and quality of life of caregivers of neurodivergent children.

## Data Availability

The datasets generated during and/or analysed during the current study are available from the corresponding author on reasonable request.
